# Myofascial trigger points: spontaneous electrical activity and its consequences for pain induction and propagation

**DOI:** 10.1186/1749-8546-6-13

**Published:** 2011-03-25

**Authors:** Hong-You Ge, César Fernández-de-las-Peñas, Shou-Wei Yue

**Affiliations:** 1 Center for Sensory-Motor Interaction (SMI), Department of Health Science and Technology, Aalborg University, Aalborg DK-9220, Denmark; 2 Department of Physical Therapy, Occupational Therapy, Rehabilitation and Physical Medicine, Universidad Rey Juan Carlos, Alcorcón, Madrid, 28922, Spain; 3 Department of Physical Medicine and Rehabilitation, Qilu Hospital, Medical School of Shandong University, Jinan 250012, PR China

## Abstract

Active myofascial trigger points are one of the major peripheral pain generators for regional and generalized musculoskeletal pain conditions. Myofascial trigger points are also the targets for acupuncture and/or dry needling therapies. Recent evidence in the understanding of the pathophysiology of myofascial trigger points supports The Integrated Hypothesis for the trigger point formation; however unanswered questions remain. Current evidence shows that spontaneous electrical activity at myofascial trigger point originates from the extrafusal motor endplate. The spontaneous electrical activity represents focal muscle fiber contraction and/or muscle cramp potentials depending on trigger point sensitivity. Local pain and tenderness at myofascial trigger points are largely due to nociceptor sensitization with a lesser contribution from non-nociceptor sensitization. Nociceptor and non-nociceptor sensitization at myofascial trigger points may be part of the process of muscle ischemia associated with sustained focal muscle contraction and/or muscle cramps. Referred pain is dependent on the sensitivity of myofascial trigger points. Active myofascial trigger points may play an important role in the transition from localized pain to generalized pain conditions *via *the enhanced central sensitization, decreased descending inhibition and dysfunctional motor control strategy.

## Introduction

Myofascial trigger points (MTPs) are hyperirritable spots in skeletal muscle associated with palpable nodules in the taut bands of muscle fibers. When these palpable nodules are stimulated mechanically, local pain and referred pain can be induced together with visible local twitch response [1,2]. MTPs can be either active or latent. An active MTP is one that refers pain either locally to a large area and/or to another remote location, the local and referred pain can be spontaneous or reproduced by mechanical stimulation which elicits a patient-recognized pain. A latent MTP does not reproduce the clinical pain complaint but may exhibit all of the features of an active MTP to a minor degree. Myofascial pain syndrome due to MTPs can be acute or chronic, regional or generalized; it can also be a primary disorder leading to local or regional pain syndromes or a secondary disorder as a consequence of other conditions [3]. Active MTPs contribute significantly to the regional acute and chronic myofascial pain syndrome [2,3], such as lateral epicondylalgia [4], headache and mechanical neck pain [5] and temporomandibular pain disorders [6]. Active MTPs are also the main peripheral pain generator in generalized musculoskeletal pain disorders [3], such as fibromyalgia and whiplash syndrome [7,8]. MTPs are the targets for acupuncture and/or dry needling [9] and other pain therapies. Indeed, MTP anesthetization decreases both pain intensity and central sensitization in local pain and generalized pain conditions [8,10,11]. Two reviews have been published recently focusing on the current state of knowledge of myofascial pain syndrome associated with MTPs [12,13]. New evidence has emerged suggesting an important role of spontaneous electrical activity (SEA) at MTPs in the induction of muscle pain and central sensitization. This article reviews the literatures in the last decade about the SEA at MTPs; in particular, how SEA contributes to the induction of local and referred pain and how active MTPs are involved in the transition from the localized pain to generalized pain conditions.

## Origin of the SEA

Registered with intramuscular needle electromyography (EMG) when the muscle is at rest, SEA is one of the characteristics of MTP [14,15]. SEA is dysfunctional extrafusal motor endplate potential (EPP) [15], rather than from the gamma motor units within muscle spindle.

Muscle tissue disruption is observed immediately after the termination of exercise, such as cytoskeletal disruptions, loss of myofibrillar registry and loss of cell integrity as manifested by intracellular plasma fibronectin stain, hypercontracted regions and invasion of inflammatory cells. In particular, muscle fiber hypercontraction occurs adjacent to fiber plasma membrane lesions and is associated with very short sarcomere lengths [16,17].

Prolonged or unaccustomed exercise, acute and chronic mechanical and electrical trauma and prolonged ischemia lead to cell membrane damage which is the initial event in muscle damage [18,19]. Following cell membrane damage, influx of Ca^2+ ^is increased, leading to Ca^2+ ^overload. As a result, calpains and phospholipase A2 may be activated; production of reactive oxygen species may be increased; and mitochondrial Ca^2+ ^may be overloaded, thereby further worsening the damage in a self-reinforcing manner [19]. In addition to Ca^2+ ^overload, an increase in Na^+ ^permeability and the accompanying increase in Na^+ ^influx also induce membrane depolarization [20]. Thus, mechanical trauma causes direct injury to the cellular membrane, causing Ca^2+ ^and Na^+ ^to flood the injured tissue. The Ca^2+ ^overload contributes to the initiation of spontaneous activity at motor endplate [21]. The localized Na^+ ^conductance change in the membrane of the active muscle fiber may also lead to the initiation of spontaneous action potentials at motor endplate [22,23]. The acetylcholine (Ach) released at a motor unit associated with MTP may be also modulated by other ion channels [24].

EPP, which is a local depolarization of the muscle fibers, spreads a short distance along the muscle fibers, with a decrement of about 50-75 per cent per millimeter. If the EPP exceeds a certain critical level (by summation of successive EPPs), endplate spikes are initiated [25], explaining the clinical phenomenon that SEA associated with MTP is registered only in a localized spot in the muscle with intramuscular needle EMG. Enormously increased abnormal spontaneous release of Ach produces the SEA. SEA is a combination of endplate noise and endplate spikes with action potentials generated by sufficient amounts of spontaneously released Ach [2,26]. Studies in MTP animal models also show that the SEA is significantly decreased by botulinum toxin which inhibits the release of acetylcholine at the neuromuscular junction [27].

**Figure 1:**
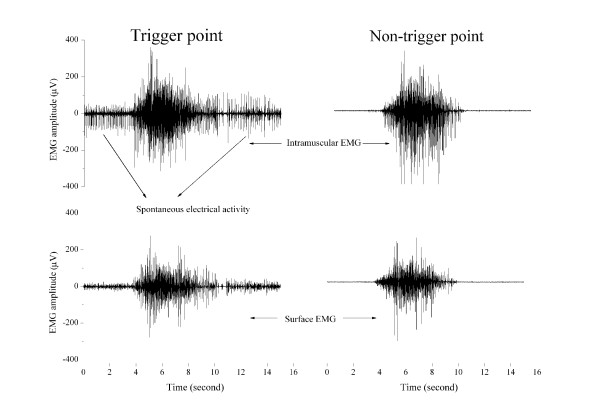
**An example of motor behavior of spontaneous electrical activity (SEA) of a myofascial trigger point (MTP) during trapezius muscle contraction**. The electromyographic (EMG) activity of the SEA of an MTP is similar to the surface EMG over an MTP on one side of the upper trapezius and to both the surface and intramuscular EMG activity of a normal muscle point on the other side of the upper trapezius. Note: following needle insertion into a MTP, surface EMG recording shows low amplitude activities.

Both extrafusal (alpha motor unit) and intrafusal fibers (gamma motor unit within muscle spindle) are cholinergically innervated; the decrease in the SEA following botulinum toxin application cannot differentiate the source of SEA from the alpha motor unit or from the gamma motor unit. The discharge patterns of static and dynamic gamma motoneurones contribute to the control of locomotion, but contraction of the intrafusal muscle fibers does not contribute to the force of muscle contraction [28]. Muscle force is positively correlated with the amplitude of EMG during dynamic contraction. Analysis of the motor behaviors of an MTP clearly shows that intramuscular EMG activity at an MTP (SEA) exhibits similar motor behavior to the surface EMG activity over an MTP and is also similar to the intramuscular and surface EMG over a non-MTP during voluntary muscle contractions in the upper trapezius muscle (Figure 1), suggesting that the SEA activity during movement contributes to the muscle force production. Thus, this motor behavior of MTP indicates that the SEA originates from the extrafusal motor endplate but not from the intrafusal motor endplate. No electrophysiological methods are currently available to record electrical activities from intrafusal motor endplate directly in the muscle. Instead, efferent discharges of intrafusal motor endplate are indirectly assessed with microneurography recorded from peripheral nerve fibers in animals and humans. Efferent discharges of intrafusal motor endplate are uncorrelated with any activation of extrafusal muscle fibers in humans [29] though intrafusal motor units are generally spontaneously active. However, the SEA may be recorded with intramuscular EMG in humans and originates from extrafusal motor endplate in several pathophysiological conditions [30], including MTPs [15]. SEA at MTPs may play a significant role in the induction of pain.

## Mechanisms of local and referred muscle pain associated with MTP

Local and referred muscle pain can be consistently induced by mechanical stimulation of active MTPs. The local and referred pain from active MTPs can be recognized by the patients as their pain experience during daily activities (activity related pain) and/or at rest (spontaneous pain) [31]. Active MTPs are responsible for patient's pain. Local and referred pain from latent MTPs are not recognized by the patients; thus latent MTPs are not responsible for patient's pain.

## Mechanisms of local pain and tenderness

Pressure pain threshold (PPT) measurement over an entire muscle shows the heterogeneous distribution (*ie *the sites with the lowest PPT corresponding to the locations of MTPs in healthy subjects), fibromyalgia [31] and chronic tension type headache [32], indicating that muscle nociceptors are sensitized at MTPs. The sensitized nociceptors lead to an increased excitability of the nociceptive nerve ending. In addition to the nociceptor sensitization, non-nociceptors (mainly the large diameter muscle afferents) are also sensitized at MTPs [33-35]; the non-nociceptors which normally do not contribute to pain perception are now involved in pain generation at MTPs. Thus, local pain and tenderness at MTPs are largely due to nociceptor sensitization with a lesser contribution from non-nociceptor sensitization.

Nociceptors and non-nociceptors sensitization at MTPs is a localized event in the muscle. The algesic substances are significantly increased at active MTP compared with latent MTP and normal muscle point [36]. These algesic substances may partly be released from the peripheral sensitized nociceptors that drive the pain associated with tissue injury [37] and may also be released from the sustained muscle fiber contraction [38,39] within muscle taut band [24]. A further study on both intramuscular and surface EMG activity recorded from an MTP for minutes revealed that the SEA was similar to a muscle cramp potential and that the increase in local muscle pain intensity was positively associated with the duration and amplitude of muscle cramp episodes [40]. The firing frequency of motor units (14.5 ± 5.1 pulses per second) during electrically-induced muscle cramp [41] is similar to that of the endplate spikes of the SEA in humans. Localized muscle cramps may induce intramuscular hypoxia, increased concentrations of algesic substances and direct mechanical stimulation of nociceptors and pain [42,43]. Human experimental studies showed that the irritability of a MTP was highly correlated with the prevalence of the SEA in the MTP as lower PPTs were associated with higher amplitude of the SEA [44]. An increased MTP sensitivity is associated with the occurrence of muscle cramps [45] and glutamate injection into a latent MTP also increases sympathetic activity with a decreased blood supply to the muscle and the skin [46]. Thus, MTP pain and tenderness is closely associated with sustained focal ischemia and focal muscle contraction and/or cramps within muscle taut band. Muscle cramps may partly underlie local and referred pain in chronic musculoskeletal pain syndromes associated with active MTPs.

## Mechanisms of referred pain from MTP

Referred pain is defined as the pain the patient feels at a remote site away from the location of an MTP. Referred pain from active MTPs is sometimes the sole complaint of patients with pain. A typical example is that patient feels pain in the front shoulder only but the pain actually comes from an active MTP in the infraspinatus.

The occurrence of referred pain is dependent on the sensitivity of an MTP. Active MTPs induce larger referred pain area and higher pain intensity than latent MTPs [31]. Experimental human pain studies also showed that the maintenance of referred pain was dependent on ongoing nociceptive input from the site of primary muscle pain [47,48]. Animal studies showed that sustained muscle damage might sensitize dorsal horn neurons and open silent synapses in adjacent segments and excite neurons that supplied the body regions in which the referred pain was felt [49]. Sustained focal ischemia and the increased algesic substances associated with muscle contraction and/or muscle cramps at MTP may sensitize the dorsal horn neurons and supraspinal structures inducing referred pain. Referred pain is a reversible process of central sensitization or neuroplasticity [50] maintained by increased peripheral nociceptive input from MTP. Inactivation of active MTP results in the disappearance of referred pain [11]. It is important to note that referred pain usually occurs seconds following mechanical stimulation of an active MTP in humans, suggesting that the induction of neuroplastic changes related to referred pain is a very rapid process, similar to the induction of central descending inhibition mechanism which is recruited a few milliseconds following intramuscular nociceptive electrical stimulation [51].

In summary, referred pain is a process of central neuroplasticity dynamically maintained by sustained nociceptive input from MTP associated with the SEA. In addition to the role in induction of local and referred pain, the SEA may also contribute to the formation of muscle taut band.

## Muscle taut band

An MTP taut band is subjectively felt by the examiner during manual palpation. Penetration of an acupuncture needle into the taut band reveals a feeling of higher resistance as compared to surrounding normal muscle tissues by the practitioners. The existence of a taut band is demonstrated by magnetic resonance elastography, indicating that the stiffness of the taut bands may be 50% greater than that of the surrounding muscle tissue [52]. Ultrasound visualization of the taut band show that MTPs appear as focal, hypoechoic regions on two-dimensional ultrasound and as focal regions of reduced vibration amplitude on vibration sonoelastography, indicating a localized, stiff nodule [53]. These findings suggest that taut bands associated with MTP are detectable and quantifiable tools for MTP diagnosis.

The mechanisms for the formation of muscle taut band are not fully understood. The molecular mechanisms of taut band formation have been detailed in a recent review [24]. SEA originates from the extrafusal motor endplate (motor unit potential) and the SEA represents focal muscle fiber contraction and/or muscle cramp. Muscle fiber contraction contributes significantly to the formation of muscle tension [54]. It is believed that this involuntary focal muscle fiber contraction and/or muscle cramps within taut muscle band contributes significantly to muscle tension and to the formation of taut band associated with MTP [24,43]. Additional contributions to the formation of taut band may come from muscle spindle afferents giving afferent signals to the extrafusal motor unit through the H-reflex pathway [33,55,56] and from the sympathetic facilitation to the SEA [57] and to MTP sensitivity [58]. Sympathetic neurotransmitter noradrenaline not only strengthens muscle tone by boosting endogenous glutamate-mediated excitation, but also transforms sub-threshold glutamatergic activity into a robust excitatory drive capable of triggering motoneurone activity [59].

Thus, muscle taut band associated with MTP may come from increased motor unit excitability with an increased release of Ach and modulated by muscle spindle afferents and sympathetic hyperactivity. One of the peripheral pain generators in the muscle, MTP may have generalized effects on the human nociceptive system.

## Role of MTPs in the transition from localized pain to generalized pain conditions

Apart from localized pain conditions, such as chronic tension type headache and migraine [5], myofascial low back pain [60], chronic prostatitis/chronic pelvic pain syndrome in men [61], lateral epicondylalgia [4], headache and mechanical neck pain [5] and temporomandibular pain disorders [6], active MTPs contribute significantly to the generalized pain conditions, such as whiplash syndrome [8] and fibromyalgia [7,10], suggesting that active MTPs play a significant role in the transition from the localized pain to generalized pain conditions. There are several ways whereby active MTPs may induce widespread pain or spatial pain propagation.

## Active MTPs induce central sensitization

Central sensitization mechanisms are involved in both the localized and generalized chronic pain conditions. Descending facilitatory and inhibitory mechanisms are involved in acute muscle nociception [62]. Persistent pain from tissue injury or inflammation contributes significantly to the induction of central sensitization and results in an enhanced net descending facilitation that contributes to the amplification and spread of pain. Mechanical stimulation or activation of latent MTPs induce mechanical hyperalgesia in extrasegmental deep tissues [40] and electrical stimulation of an active MTPs significantly enhance somatosensory and limbic activity in the brain [63]. Inactivation of active MTPs with consecutive anesthetic injections significantly decreases mechanical hyperalgesia and/or allodynia and referred pain in both localized pain condition of migraine [11] and generalized pain conditions of fibromyalgia [10] and whiplash syndrome [8]. Thus, active MTPs are one of the sources of peripheral nociceptive input inducing central sensitization.

Central sensitization may increase the MTP sensitivity through segmental pathways resulting in decreased mechanical pain threshold [64] and increased amplitude of the SEA [65]. The influence of a central MTP on satellite MTPs may play a significant role in the segmental pain propagation in chronic generalized pain conditions; however, no evidence supports that central sensitization can induce the development of new MTPs. Further studies are needed to investigate the relationship between central sensitization and the MTP formation.

## Active MTPs impair descending inhibition

In chronic musculoskeletal pain conditions, the balance between supraspinal facilitation and inhibition of pain shifts towards an overall decrease in inhibition. Muscle pain impairs diffuse noxious inhibitory control mechanisms [66]. Inactivation of active MTPs with ultrasound and dry needling temporarily increases mechanical pain threshold in local pain syndromes [67,68]. Inactivation of active MTPs results in an increased mechanical pain threshold in fibromyalgia patients [10]. Active MTPs are one of the major contributors to the impaired descending inhibition in chronic musculoskeletal pain conditions. Impaired descending inhibition in chronic musculoskeletal pain conditions, which is same as an enhanced central sensitization, leads to an increased mechanical pain sensitivity of muscle tissue (*ie *muscle becomes more tender upon mechanical stimulation). Related to this mechanism, PPT at latent MTPs located in various body parts may become lower; latent MTPs are easily activated in response to various perpetuating factors. Pain propagation may thus be observed in the segmental and/or extrasegmental muscles in generalized chronic pain conditions.

## Active MTPs impair motor control strategy

Upper trapezius muscle is active across the duration of shoulder activities and the frequency of differential activation between cranial and caudal regions within the upper trapezius is lower in fibromyalgia patients than controls [69,70]. Sustained muscle activation induces muscle ischemia [71] and increases the release of algesic substances in the muscle and cytokines in the blood [39,72] and eventually decreases the muscle mechanical pain threshold more in the cranial region than the caudal region. Sustained muscle contraction at low load levels may damage muscle tissues and increase MTP sensitivity and latent MTPs may be activated and result in local and referred pain. An increased muscle co-activation has also been observed in local pain conditions, such as tension type headache [73]. An increased co-activation of antagonist musculature may reflect reorganization of the motor control strategy in patients, potentially leading to muscle overload and increased nociception. While active MTPs are present in these patients, there is no direct evidence on whether the impaired motor control strategy is associated with the existence of active MTPs. However, latent MTPs are associated with impaired motor activation pattern and the elimination of these latent MTPs induces normalization of the impaired motor activation pattern [74,75]. The impaired motor control strategy may partially underlie the induction of local pain and segmental pain propagation.

## Conclusion

SEA at the MTP arises from the extrafusal motor endplate, representing focal muscle fiber contraction and/or muscle cramp potentials within taut band. The sustained focal muscle fiber contraction and/or muscle cramp potentials contribute to the induction of local and referred pain. Active MTPs may play an important role in the transition from the localized pain to generalized pain conditions *via *the enhanced central sensitization, decreased descending inhibition and dysfunctional motor control strategy.

## Abbreviations

Ach: acetylcholine; EMG: electromyography; EPP: endplate potential; MTP: myofascial trigger point; PPT: pressure pain threshold; SEA: spontaneous electrical activity;

## Competing interests

The authors declare that they have no competing interests.

## Authors' contributions

HYG did the literature search. HYG, CFP and SWY jointly drafted the manuscript. HYG revised the manuscript. All authors read and approved the final version of the manuscript.

## References

[B1] Gerwin RD Dommerholt J Shah JP An expansion of Simons' integrated hypothesis of trigger point formation Curr Pain Headache Rep 2004 8 468 475 10.1007/s11916-004-0069-x 15509461

[B2] Simons DG Review of enigmatic MTrPs as a common cause of enigmatic musculoskeletal pain and dysfunction J Electromyogr Kinesiol 2004 14 95 107 10.1016/j.jelekin.2003.09.018 14759755

[B3] Gerwin RD Classification, epidemiology, and natural history of myofascial pain syndrome Curr Pain Headache Rep 2001 5 412 420 10.1007/s11916-001-0052-8 11560806

[B4] Fernández-Carnero J Fernández-de-las-Peñas C de la Llave-Rincón AI Ge HY Arendt-Nielsen L Bilateral myofascial trigger points in the forearm muscles in patients with chronic unilateral lateral epicondylalgia: a blinded, controlled study Clin J Pain 2008 24 802 807 18936598 10.1097/AJP.0b013e31817bcb79

[B5] Fernandez-de-Las-Penas C Simons D Cuadrado ML Pareja J The role of myofascial trigger points in musculoskeletal pain syndromes of the head and neck Curr Pain Headache Rep 2007 11 365 372 10.1007/s11916-007-0219-z 17894927

[B6] Fernández-de-Las-Peñas C Galán-del-Río F Alonso-Blanco C Jiménez-García R Arendt-Nielsen L Svensson P Referred pain from muscle trigger points in the masticatory and neck-shoulder musculature in women with temporomandibular disoders J Pain 2010 11 1295 1304 20494623 10.1016/j.jpain.2010.03.005

[B7] Ge HY Prevalence of myofascial trigger points in fibromyalgia: the overlap of two common problems Curr Pain Headache Rep 2010 14 339 345 10.1007/s11916-010-0127-5 20607459

[B8] Freeman MD Nystrom A Centeno C Chronic whiplash and central sensitization; an evaluation of the role of a myofascial trigger points in pain modulation J Brachial Plex Peripher Nerve Inj 2009 4 2 10.1186/1749-7221-4-2 19389231 PMC2680858

[B9] Moral OMD Dry needling treatments for myofascial trigger points J Musculoskelet Pain 2010 18 411 416 10.3109/10582452.2010.502632

[B10] Affaitati G Costantini R Fabrizio A Lapenna D Tafuri E Giamberardino MA Effects of treatment of peripheral pain generators in fibromyalgia patients Eur J Pain 2011 15 61 69 10.1016/j.ejpain.2010.09.002 20889359

[B11] Giamberardino MA Tafuri E Savini A Fabrizio A Affaitati G Lerza R Di Ianni L Lapenna D Mezzetti A Contribution of myofascial trigger points to migraine symptoms J Pain 2007 8 869 878 10.1016/j.jpain.2007.06.002 17690015

[B12] Gerwin R Myofascial pain syndrome: Here we are, where must we go J Musculoskelet Pain 2010 18 329 347 10.3109/10582452.2010.502636

[B13] Kuan T Current studies on myofascial pain syndrome Curr Pain Headache Rep 2009 13 365 369 10.1007/s11916-009-0059-0 19728962

[B14] Hubbard DR Berkoff GM Myofascial trigger points show spontaneous needle EMG activity Spine 1993 18 1803 1807 10.1097/00007632-199310000-00015 8235865

[B15] Simons DG Hong CZ Simons LS Endplate potentials are common to midfiber myofacial trigger points Am J Phys Med Rehabil 2002 81 212 222 10.1097/00002060-200203000-00010 11989519

[B16] Fridén J Lieber RL Segmental muscle fiber lesions after repetitive eccentric contractions Cell Tissue Res 1998 293 165 171 9634608 10.1007/s004410051108

[B17] Duncan CJ Jackson MJ Different mechanisms mediate structural changes and intracellular enzyme efflux following damage to skeletal muscle J Cell Sci 1987 87 183 188 3117809 10.1242/jcs.87.1.183

[B18] Armstrong RB Warren GL Warren JA Mechanisms of exercise-induced muscle fibre injury Sports Med 1991 12 184 207 10.2165/00007256-199112030-00004 1784873

[B19] Gissel H The role of Ca2 in muscle cell damage Ann N Y Acad Sci 2006 1066 166 180 10.1196/annals.1363.013 16533926

[B20] McBride TA Stockert BW Gorin FA Carlsen RC Stretch-activated ion channels contribute to membrane depolarization after eccentric contractions J Appl Physiol 2000 88 91 101 10642367 10.1152/jappl.2000.88.1.91

[B21] Katz B Miledi R Spontaneous and evoked activity of motor nerve endings in calcium Ringer J Physiol 1969 203 689 706 4318717 10.1113/jphysiol.1969.sp008887 PMC1351538

[B22] Purves D Sakmann B Membrane properties underlying spontaneous activity of denervated muscle fibres J Physiol 1974 239 125 153 4853156 10.1113/jphysiol.1974.sp010559 PMC1330941

[B23] Harvey PJ Li Y Li X Bennett DJ Persistent sodium currents and repetitive firing in motoneurons of the sacrocaudal spinal cord of adult rats J Neurophysiol 2006 96 1141 1157 10.1152/jn.00335.2005 16282206 PMC5726388

[B24] Gerwin RD The taut band and other mysteries of the trigger point: An examination of the mechanisms relevant to the development and maintenance of the trigger point J Musculoskelet Pain 2008 16 115 121 10.1080/10582450801960081

[B25] Eccles JC Katz B Kuffler SW Nature of the" endplate potential" in curarized muscle J Neurophysiol 1941 4 362 387

[B26] Heuser J Miledi R Effect of lanthanum ions on function and structure of frog neuromuscular junctions Proc R Soc Lond B Biol Sci 1971 179 247 260 10.1098/rspb.1971.0096 4400214

[B27] Kuan TS Chen JT Chen SM Chien CH Hong CZ Effect of botulinum toxin on endplate noise in myofascial trigger spots of rabbit skeletal muscle Am J Phys Med Rehabil 2002 81 512 520 10.1097/00002060-200207000-00008 12131178

[B28] Ellaway P Taylor A Durbaba R Rawlinson S Role of the fusimotor system in locomotion Adv Exp Med Biol 2002 508 335 342 12171129 10.1007/978-1-4615-0713-0_39

[B29] Ribot E Roll J Vedel J Efferent discharges recorded from single skeletomotor and fusimotor fibres in man J Physiol 1986 375 251 3795058 10.1113/jphysiol.1986.sp016115 PMC1182757

[B30] Buchthal F Spontaneous electrical activity: an overview Muscle Nerve 1982 5 S52 9 10.1002/mus.880050403 6763149

[B31] Ge HY Nie H Madeleine P Danneskiold-Samsoe B Graven-Nielsen T Arendt-Nielsen L Contribution of the local and referred pain from active myofascial trigger points in fibromyalgia syndrome Pain 2009 147 233 240 10.1016/j.pain.2009.09.019 19819074

[B32] Fernández-de-las-Peñas C Caminero AB Madeleine P Guillem-Mesado A Ge HY Arendt-Nielsen L Pareja JA Multiple active myofascial trigger points and pressure pain sensitivity maps in the temporalis muscle are related in women with chronic tension type headache Clin J Pain 2009 25 506 512 19542799 10.1097/AJP.0b013e3181a08747

[B33] Ge HY Serrao M Andersen OK Graven-Nielsen T Arendt-Nielsen L Increased H-reflex response induced by intramuscular electrical stimulation of latent myofascial trigger points Acupunct Med 2009 27 150 154 10.1136/aim.2009.001099 19942720

[B34] Li LT Ge HY Yue SW Arendt-Nielsen L Nociceptive and non-nociceptive hypersensitivity at latent myofascial trigger points Clin J Pain 2009 25 132 137 10.1097/AJP.0b013e3181878f87 19333159

[B35] Wang YH Ding XL Zhang Y Chen J Ge HY Arendt-Nielsen L Yue SW Ischemic compression block attenuates mechanical hyperalgesia evoked from latent myofascial trigger points Exp Brain Res 2010 202 265 270 10.1007/s00221-009-2129-2 20035322

[B36] Shah JP Danoff JV Desai MJ Parikh S Nakamura LY Phillips TM Gerber LH Biochemicals associated with pain and inflammation are elevated in sites near to and remote from active myofascial trigger points Arch Phys Med Rehabil 2008 89 16 23 10.1016/j.apmr.2007.10.018 18164325

[B37] Willis W Jr Dorsal root potentials and dorsal root reflexes: a double-edged sword Exp Brain Res 1999 124 395 421 10.1007/s002210050637 10090653

[B38] Tegeder I Zimmermann J Meller S Geisslinger G Release of algesic substances in human experimental muscle pain Inflamm Res 2002 51 393 402 10.1007/PL00000320 12234056

[B39] Rosendal L Larsson B Kristiansen J Peolsson M Søgaard K Kjær M Sørensen J Gerdle B Increase in muscle nociceptive substances and anaerobic metabolism in patients with trapezius myalgia: microdialysis in rest and during exercise Pain 2004 112 324 334 10.1016/j.pain.2004.09.017 15561388

[B40] Xu YM Ge HY Arendt-Nielsen L Sustained nociceptive mechanical stimulation of latent myofascial trigger point induces central sensitization in healthy subjects J Pain 2010 11 1348 1355 20451466 10.1016/j.jpain.2010.03.010

[B41] Minetto MA Holobar A Botter A Farina D Discharge properties of motor units of the abductor hallucis muscle during cramp contractions J Neurophysiol 2009 102 1890 1901 10.1152/jn.00309.2009 19571196

[B42] Laferriere A Millecamps M Xanthos D Xiao W Siau C de Mos M Sachot C Ragavendran JV Huygen F Bennett G Coderre T Cutaneous tactile allodynia associated with microvascular dysfunction in muscle Mol Pain 2008 4 49 18957097 10.1186/1744-8069-4-49 PMC2584041

[B43] Simons D Mense S Understanding and measurement of muscle tone as related to clinical muscle pain Pain 1998 75 1 17 10.1016/S0304-3959(97)00102-4 9539669

[B44] Kuan TS Hsieh YL Chen SM Chen JT Yen WC Hong CZ The myofascial trigger point region: correlation between the degree of irritability and the prevalence of endplate noise Am J Phys Med Rehabil 2007 86 183 189 10.1097/PHM.0b013e3180320ea7 17314703

[B45] Ge HY Zhang Y Boudreau S Yue SW Arendt-Nielsen L Induction of muscle cramps by nociceptive stimulation of latent myofascial trigger points Exp Brain Res 2008 187 623 629 10.1007/s00221-008-1331-y 18317742

[B46] Zhang Y Ge HY Yue SW Kimura Y Arendt-Nielsen L Attenuated skin blood flow response to nociceptive stimulation of latent myofascial trigger points Arch Phys Med Rehabil 2009 90 325 332 10.1016/j.apmr.2008.06.037 19236988

[B47] Rubin TK Gandevia SC Henderson LA Macefield VG Effects of intramuscular anesthesia on the expression of primary and referred pain induced by intramuscular injection of hypertonic saline J Pain 2009 10 829 835 19380258 10.1016/j.jpain.2009.01.327

[B48] Graven-Nielsen T Arendt-Nielsen L Svensson P Jensen TS Quantification of local and referred muscle pain in humans after sequential im injections of hypertonic saline Pain 1997 69 111 117 10.1016/S0304-3959(96)03243-5 9060020

[B49] Hoheisel U Koch K Mense S Functional reorganization in the rat dorsal horn during an experimental myositis Pain 1994 59 111 118 10.1016/0304-3959(94)90054-X 7854791

[B50] Arendt-Nielsen L Laursen RJ Drewes AM Referred pain as an indicator for neural plasticity Prog Brain Res 2000 129 343 356 full_text 11098702 10.1016/s0079-6123(00)29026-2

[B51] Ge HY Collet T Mørch CD Arendt-Nielsen L Andersen OK Depression of the human nociceptive withdrawal reflex by segmental and heterosegmental intramuscular electrical stimulation Clin Neurophysiol 2007 118 1626 1632 10.1016/j.clinph.2007.04.007 17507291

[B52] Chen Q Bensamoun S Basford JR Thompson JM An KN Identification and quantification of myofascial taut bands with magnetic resonance elastography Arch Phys Med Rehabil 2007 88 1658 1661 10.1016/j.apmr.2007.07.020 18047882

[B53] Sikdar S Shah JP Gebreab T Yen RH Gilliams E Danoff J Gerber LH Novel applications of ultrasound technology to visualize and characterize myofascial trigger points and surrounding soft tissue Arch Phys Med Rehabil 2009 90 1829 1838 10.1016/j.apmr.2009.04.015 19887205 PMC2774893

[B54] Rosales RL Arimura K Takenaga S Osame M Extrafusal and intrafusal muscle effects in experimental botulinum toxin-A injection Muscle Nerve 1996 19 488 496 10.1002/(SICI)1097-4598(199604)19:4<488::AID-MUS9>3.0.CO;2-8 8622728

[B55] Cummings M Myofascial trigger points: does recent research gives new insights into the pathophysiology? Acupunct Med 2009 27 148 149 10.1136/aim.2009.001289 19942719

[B56] Chaix Y Marque P Meunier S Pierrot-Deseilligny E Simonetta-Moreau M Further evidence for non-monoynaptic group I excitation of motoneurones in the human lower limb Exp Brain Res 1997 115 35 46 10.1007/PL00005683 9224832

[B57] Chen JT Chen SM Kuan TS Chung KC Hong CZ Phentolamine effect on the spontaneous electrical activity of active loci in a myofascial trigger spot of rabbit skeletal muscle Arch Phys Med Rehabil 1998 79 790 794 10.1016/S0003-9993(98)90357-4 9685092

[B58] Ge HY Fernández-de-las-Peñas C Arendt-Nielsen L Sympathetic facilitation of hyperalgesia evoked from myofascial tender and trigger points in patients with unilateral shoulder pain Clin Neurophysiol 2006 117 1545 1550 10.1016/j.clinph.2006.03.026 16737848

[B59] Schwarz PB Yee N Mir S Peever JH Noradrenaline triggers muscle tone by amplifying glutamate-driven excitation of somatic motoneurones in anaesthetized rats J Physiol 2008 586 5787 5802 10.1113/jphysiol.2008.159392 18845613 PMC2655409

[B60] Itoh K Katsumi Y Kitakoji H Trigger point acupuncture treatment of chronic low back pain in elderly patients-a blinded RCT Acupunct Med 2004 22 170 177 10.1136/aim.22.4.170 15628774

[B61] Anderson RU Sawyer T Wise D Morey A Nathanson BH Krieger JN Painful myofascial trigger points and pain sites in men with chronic prostatitis/chronic pelvic pain syndrome J Urol 2009 182 2753 2758 10.1016/j.juro.2009.08.033 19837420

[B62] You HJ Lei J Sui MY Huang L Tan YX Tjolsen A Arendt-Nielsen L Endogenous descending modulation: spatiotemporal effect of dynamic imbalance between descending facilitation and inhibition of nociception J Physiol 2010 588 4177 4188 10.1113/jphysiol.2010.196923 20837643 PMC3002449

[B63] Niddam DM Chan RC Lee SH Yeh TC Hsieh JC Central representation of hyperalgesia from myofascial trigger point Neuroimage 2008 39 1299 1306 10.1016/j.neuroimage.2007.09.051 17999939

[B64] Srbely JZ Dickey JP Bent LR Lee D Lowerison M Capsaicin-induced central sensitization evokes segmental increases in trigger point sensitivity in humans J Pain 2010 11 636 643 10.1016/j.jpain.2009.10.005 20015704

[B65] Fernandez-Carnero J Ge HY Kimura Y Fernandez-de-Las-Penas C Arendt-Nielsen L Increased spontaneous electrical activity at a latent myofascial trigger point after nociceptive stimulation of another latent trigger point Clin J Pain 2010 26 138 143 10.1097/AJP.0b013e3181bad736 20090441

[B66] Arendt-Nielsen L Sluka KA Nie HL Experimental muscle pain impairs descending inhibition Pain 2008 140 465 471 10.1016/j.pain.2008.09.027 18977598 PMC2732020

[B67] Srbely JZ Dickey JP Lowerison M Edwards AM Nolet PS Wong LL Stimulation of myofascial trigger points with ultrasound induces segmental antinociceptive effects: A randomized controlled study Pain 2008 139 260 266 10.1016/j.pain.2008.04.009 18508198

[B68] Srbely JZ Dickey JP Lee D Lowerison M Dry needle stimulation of myofascial trigger points evokes segmental anti-nociceptive effects J Rehabil Med 2010 42 463 468 10.2340/16501977-0535 20544158

[B69] Falla D Andersen H Danneskiold-Samsøe B Arendt-Nielsen L Farina D Adaptations of upper trapezius muscle activity during sustained contractions in women with fibromyalgia J Electromyogr Kinesiol 2010 20 457 464 10.1016/j.jelekin.2009.07.002 19682926

[B70] Gerdle B Gronlund C Karlsson S Holtermann A Roeleveld K Altered neuromuscular control mechanisms of the trapezius muscle in fibromyalgia BMC Musculoskelet Disord 2010 11 42 10.1186/1471-2474-11-42 20205731 PMC2839982

[B71] Elvin A Siosteen AK Nilsson A Kosek E Decreased muscle blood flow in fibromyalgia patients during standardised muscle exercise: A contrast media enhanced colour doppler study Eur J Pain 2006 10 137 144 10.1016/j.ejpain.2005.02.001 16310717

[B72] Ross RL Jones KD Bennett RM Ward RL Druker BJ Wood LJ Preliminary evidence of increased pain and elevated cytokines in fibromyalgia patients with defective growth hormone response to exercise Open Immunol J 2010 3 9 18 20467575 10.2174/1874226201003010009 PMC2868257

[B73] Fernandez-de-las-Peñas C Falla D Arendt-Nielsen L Farina D Cervical muscle co-activation in isometric contractions is enhanced in chronic tension-type headache patients Cephalalgia 2008 28 744 751 18460003 10.1111/j.1468-2982.2008.01584.x

[B74] Lucas KR Rich PA Polus BI Muscle activation patterns in the scapular positioning muscles during loaded scapular plane elevation: the effects of latent myofascial trigger points Clin Biomech (Bristol, Avon) 2010 25 765 770 10.1016/j.clinbiomech.2010.05.006 20667633

[B75] Lucas KR The impact of latent trigger points on regional muscle function Curr Pain Headache Rep 2008 12 344 349 10.1007/s11916-008-0058-6 18765139

